# Regionally differentiated promotion of electric vehicles in China considering environmental and human health impacts

**DOI:** 10.1088/1748-9326/acdbde

**Published:** 2023-06-22

**Authors:** Yan Ru Fang, Xin Sun, Silu Zhang, Gang Liu, Xiaorui Liu, Peng Zhang, Yifei Kang, Hancheng Dai

**Affiliations:** 1 College of Environmental Sciences and Engineering, Peking University, Beijing 100871, People’s Republic of China; 2 China Automotive Technology and Research Center Co., Ltd, No. 68, East Xianfeng Road, Dongli District, Tianjin 300300, People’s Republic of China; 3 Institute for Global Health and Development, Peking University, Beijing 100871, People’s Republic of China; 4 College of Urban and Environmental Sciences, Peking University, Beijing 100871, People’s Republic of China; 5 Automotive Data of China (Tianjin) Co., Ltd, No. 3 Wanhui Road, Zhongbei Town, Xiqing District, Tianjin 300393, People’s Republic of China; 6 Automotive Data of China Co., Ltd, Boxing 6th Road, Beijing Economic Development Zone, Beijing 100176, People’s Republic of China; 7 Beijing Yiwei New Energy Vehicles Big Data Application &Technology Research Center, 2 North Xisanhuan Road, Haidian District, Beijing 100081, People’s Republic of China

**Keywords:** carbon neutrality, co-benefits, electric vehicle, health impact, IMED model, retrospective analysis

## Abstract

Private passenger vehicles, with its high emissions of CO_2_ and air pollutants, poses a severe threat to global climate and human health, particularly for a large developing country like China. Although both energy efficiency improvement of internal combustion engine vehicles (ICEVs) and the wide adoption of electric vehicles (EVs) could contribute to reducing emissions, how they should be jointly implemented in provinces with a heterogeneous context to maximize their net benefits remains insufficiently explored. Here, based on an integrated modeling framework associated with one factual (REF) and four counterfactual scenarios to explore the priority and best-ranked ordering of both EVs’ penetration and high energy-efficient ICEVs in 31 Chinese provinces to achieve the most environmental and human health benefits from 2011 to 2018. The results demonstrate that electrification of the passenger fleet, which is charged by a slightly cleaner power source relative to 2011, yields significant co-benefits of CO_2_ reduction and air quality improvement. Compared with REF, the fleet electrification scenario would lead to 3167 cases of avoided mortality and attain US$4.269 billion of health benefits in 2018, accounting for 0.03% of China’s gross domestic product. Nonetheless, highly efficient ICEVs are found to harbor decarbonization potential and health benefits in northern China. Based on these results, Sichuan, Hebei and seven other provinces in east China should promote EVs imminently; conversely, eight provinces with a high share of thermal power must continually advance their implementation of ICEVs in the near future. Such prioritization of EVs and ICEV development at the provincial level provides timely insights for devising tailored policies regarding passenger car transition and for maximizing climate and health benefits based on regional heterogeneity.

## 1. Introduction

The transportation sector is recognized as a pivotal bottleneck to achieving global sustainability due to its essential role in deep decarbonization (Creutzig *et al* 2015) and pollution mitigation (Colvile *et al* 2001). For example, the transportation sector accounted for 24.5% of the world’s total carbon dioxide (CO_2_) emissions in 2019 (IEA 2021b). Meanwhile, China accounted for 10% of the global transportation CO_2_ emissions (IEA 2021b), more than 80% of which comes from road transportation (IEA 2019), particularly passenger transport (Zhang and Nian 2013, Hao *et al* 2014, Zhao *et al* 2019). Moreover, private vehicles (PVs) contribute significantly to air pollutant emissions (Wu *et al* 2017, Chen *et al* 2020, MEE 2021), notably in the form of ambient fine particulate matter with an aerodynamic diameter of ⩽2.5 *μ*m (PM_2.5_), nitrogen oxide (NO*_X_*), sulfur dioxide (SO_2_), as well as volatile organic compounds (VOCs) (Zhao *et al* 2013, He *et al* 2016).

Electric vehicles (EVs) are currently the most widely discussed and sought-after solution worldwide for lowering CO_2_ emissions and operational cost savings (Orsi *et al* 2016). Moreover, EV penetration also provides co-benefits for reducing emissions of air pollutants (Huo *et al* 2015, Bellocchi *et al* 2018, Alimujiang and Jiang 2020, Ou *et al* 2021, Schnell *et al* 2021). Therefore, China’s national and provincial governments have focused on devising various subsidies and infrastructural arrangements to encourage EVs’ adoption and implementation (table S1). EV technologies are usually composed of plug-in hybrid electric vehicles (PHEVs) and battery electric vehicles (BEVs), while in a broader sense, PHEVs, BEVs, hybrid electric vehicles (HEVs), and fuel cell vehicles (FCVs) are also classified as electrified vehicles. In 2019, when China overtook the USA to become the most prominent automotive market and producer, the share of EVs in its total vehicle fleet rose to 2%, higher than the global average of 1% (IEA 2021a).

A burgeoning body of literature has tried to assess the climate and/or health impacts of the passenger car transition in China. For example, Ou *et al* (2010) analyzed the vehicle stock dynamics and associated energy demand and greenhouse gas (GHG) emissions in the road transport sector (including EVs’ implementation) through 2050 in China. The results concluded that highly efficient EVs should be supported in future policy implementation to achieve decarbonization of transportation. Hao *et al* (2015) established a bottom-up accounting framework to estimate the energy consumption and GHG emissions from China’s PVs based on a set of scenarios till 2050. It was found that BEVs coupled with low-emission electricity could achieve a deep cut in CO_2_ emissions. Both studies were undermined by technological uncertainties and did not consider the specific vehicles’ traveled distance. Moreover, the current electricity mix of China is still dominated by thermal power (Zhuo *et al* 2022). Therefore, the actual climate and health benefits of EVs’ penetration may be offset from a life cycle perspective (e.g. a trade-off in emissions between the transport and electricity sectors) (Gryparis *et al* 2020). For example, a previous study pointed out that EVs could drastically increase SO_2_ and NO*_x_* emissions under the current electricity grid compared to gasoline-powered vehicles (Huo *et al* 2010).

Furthermore, the regional heterogeneity in the electric mix, level of socio-economic development, fleet status, and transition pathways would be significant for determining the extent of climate and health benefits associated with car electrification across a huge country like China. For example, Wu *et al* (2019) studied the interprovincial variation in CO_2_ emissions for EVs and internal combustion engine vehicles (ICEVs) according to their size segments in China. They proposed that the small size segments of BEVs should be encouraged because of a high ratio of thermal electricity. Ke *et al* (2017) considered passenger vehicle electrification in China’s Yangtze River Delta region and found that EVs could improve air quality at both regional and urban levels. Li *et al* (2021) employed a well-to-wheel model that considers regional differences in the electricity mix to reveal the potential to reduce SO_2_ and NO*_X_* emissions associated with EV adoption. However, the varying electricity mix for EVs’ adoption and energy efficiency of existing ICEVs among provinces also suggests the importance of balancing the energy efficiency improvement of existing ICEVs against the further adoption of EVs in differing provinces for maximizing the total emission reduction there. Indeed, other studies reported that energy efficiency improvements for ICEVs can also benefit carbon emission reduction (Ahman 2001, Gabriel-Buenaventura and Azzopardi 2015, Su *et al* 2018).

Due to the disparity of economic development, population dynamics, the current status of EV penetration, vehicle stock and power mix in China’s 31 provincial regions, the EV and ICEV transition paths may vary at the provincial level. However, to our best knowledge, the combined effects of emission reduction, air quality improvement, human health and health benefits of EVs’ penetration in a currently thermal power-dominated electricity mix in different provinces have not been explicitly explored in any previous studies. Without further considering the electricity mix, diffusion of the EV may increase carbon and air pollutants emissions and induce devastating human health impacts. Consequently, a strategy for EV promotion heterogeneity at the provincial level is necessary.

To address the abovementioned knowledge gaps, we integrated China’s historical data from 2011 to 2018 into an integrated model for a retrospective analysis. We designed a set of counterfactual scenarios to identify the impacts of EVs’ and ICEVs’ development on CO_2_ emissions, air pollutants, air quality, human health and health benefits in 31 Chinese provinces. The model results could help achieve maximum emission reductions, air quality improvement, and health benefits for ranking the gradient development order of EV promotion and provide timely insights that can inform a tailored policy approach for climate change mitigation of the transport sector, both nationally and regionally.

## 2. Materials and methods

### 2.1. Study framework

**Figure 1.**
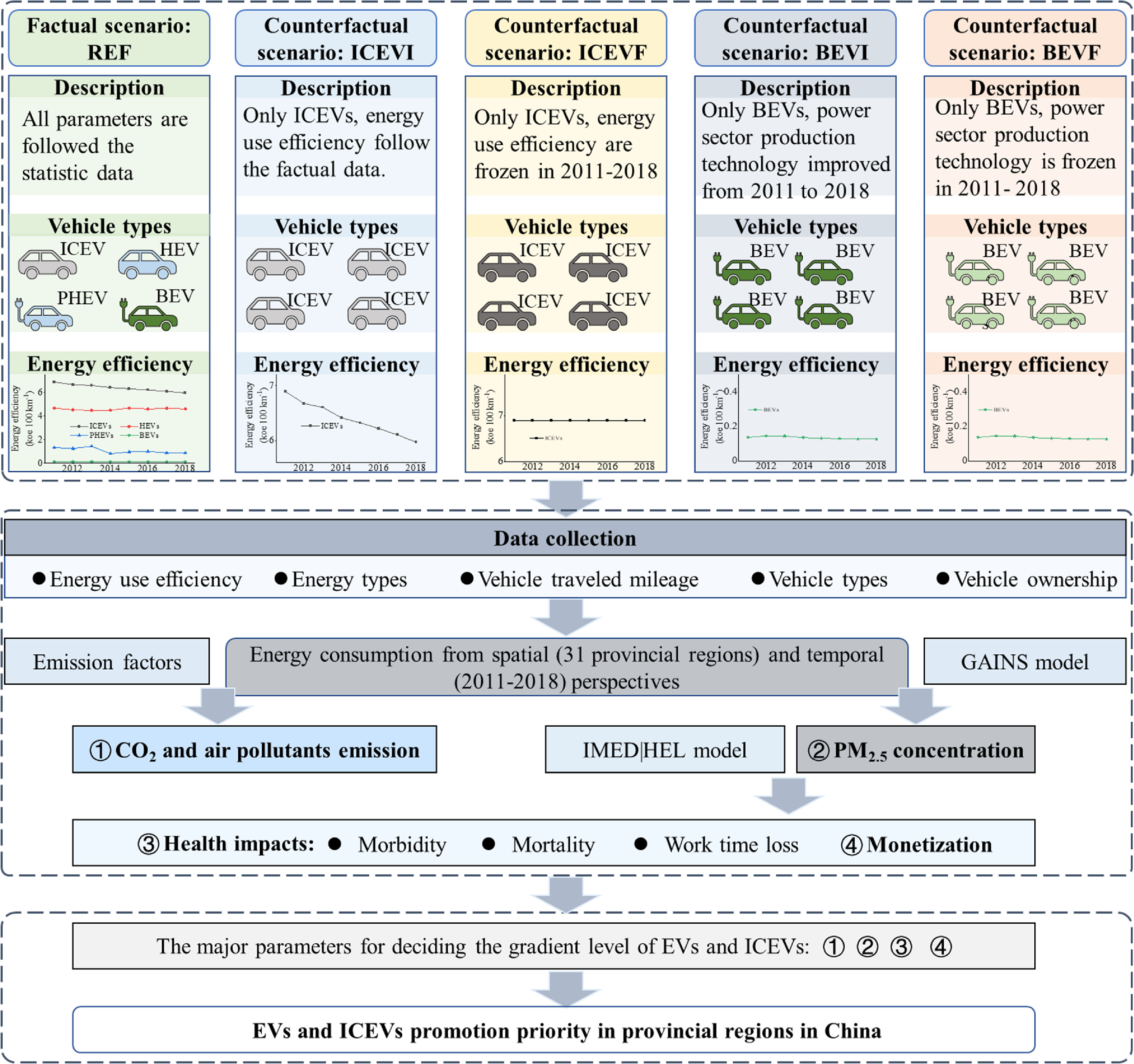
The integrated modeling framework for assessing the vehicle emissions and health impacts of different transportation transition scenarios at the province level.

The present study adopted an integrated modeling approach that combines CO_2_ emissions, air quality levels, and health assessments under different fleet electrification scenarios for the 31 provincial regions (i.e. provinces, municipalities directly under the central government, and autonomous regions, as depicted in figure S1 in the supporting information) of China (figure 1). Specifically, we explored the emission changes of CO_2_ and four major air pollutants—PM_2.5_, SO_2_, NO*x*, and VOCs—in 31 provinces from 2011 to 2018 in China. Air quality was generated by a Global Air Pollution Information and Simulation (GAINS) model, whereby GAINS was used to estimate the ambient concentration of PM_2.5_ among five scenarios. The health impact evaluation model assessed the health effects of human exposure PM_2.5_ under different scenarios. The adverse health outcomes included mortality, morbidity, and loss of work time, while the health benefits entailed monetary values of illness and the value of a statistical life. Finally, according to the above results, we determined an order to prioritize the development of EVs and ICEVs in 31 provincial regions incorporating their regional heterogeneity.

The latest data on PV sales volume, vehicle ownership, and energy efficiency were obtained from China Automotive Technology and Research Center Co., Ltd. This data covers all the PVs in China from 2011 to 2018. The annual mileage for vehicle travel for the 31 provincial regions was collected from the National Big Data Alliance of New Energy Vehicles (Wang 2020).

### 2.2. Scenario design

This study set up five scenarios for CO_2_ and air pollutant emissions and the health benefits analysis (figure 1). The first scenario is a reference case coupled with four counterfactual scenarios for further research. This reference scenario (REF) was designed based on historical data, corresponding to PVs’ technical structure and energy efficiency following the trend of actual changes. Types of PVs include ICEVs, HEVs, PHEVs, and BEVs, and the technology mix of power generation follows the historical development trend of 2011–2018 under this scenario. **ICEVI** denotes an ‘**I**nternal **c**ombustion **e**ngine **v**ehicles with **i**mproving energy efficiency’ scenario; it is a counterfactual scenario representing energy efficiency undergoing an improved trend for ICEVs from 2011 to 2018, in which all PVs become ICEVs and the energy efficiency follows the actual data. **ICEVF** denotes an ‘**I**nternal **c**ombustion **e**ngine **v**ehicles with **f**rozen energy efficiency’ scenario; it also supposed all PVs become ICEVs, but their energy efficiency is frozen from 2011 till 2018 (fixed at 2011 levels). BEVI denotes the ‘**B**attery **e**lectric **v**ehicles with **i**mproved electricity’ scenario; it assumes all vehicles in this scenario are BEVs. The power sector follows the factual trends of a decreasing share of thermal electricity, the same as for REF. Finally, BEVF denotes the ‘**B**attery **e**lectric **v**ehicles with **f**rozen electricity’ scenario; this has the same design as the BEVI scenario regarding vehicle structure; all PVs are presumed to be BEVs. But the power generation technology did not change from 2011 to 2018.

Thus, the difference between ICEVI and REF results would show the effects of ICEVs altered by small-scale electrification (<2.5%). Correspondingly, the difference between ICEVI and ICEVF would demonstrate the impact of improving the energy efficiency of ICEVs. Moreover, by comparing the results of BEVI and REF, the effects of small-scale electrification transfer to all BEVs could be gleaned. The discrepancies between BEVI versus BEVF scenarios can indicate the impact of a slightly cleaner power source (an average of 70% thermal power in China in 2018).

### 2.3. Accounting for energy consumption and emissions of CO_2_ and air pollutants

Energy consumption in this study is calculated by multiplying three quantified terms: energy efficiency per vehicle per kilometer, vehicle ownership, and vehicle traveled miles (equation (1)).

\begin{equation*}{\text{EC = EE}} \times {\text{VO}} \times {\text{VMT}}\end{equation*}

where, EC is the total energy consumption from different types of PVs under various scenarios from 2011 to 2018 in the 31 provincial regions (toe); EE is energy efficiency (toe km^−1^); VO is the vehicle ownership, and VMT is the vehicle traveled miles (in km).

The emission factor of CO_2_ for gasoline came from the GAINS model. The Multi-Resolution Emission Inventory for China calculated the electricity emission factor. The respective emission factors of four air pollutants were derived based on vehicle classification (ICEVs, HEVs, PHEVs, and BEVs), fuel type (gasoline, electricity), and emission standard level (from Pre-State I to State V). We also obtained data from the *On-road Vehicle Air Pollutants Emissions Inventory Guidebook* (MEE 2014) and relevant published research studies (Feng and Xu 2016, Sun *et al* 2019).

### 2.4. Evaluation of air quality and health impacts

The GAINS model simulated PM_2.5_ concentrations under different scenarios. The Integrated Model of Economy, Energy and Environment for Sustainable Development|Health (IMED|HEL) was used here to evaluate the health impacts of PM_2.5_ exposure. The PM_2.5_ concentration-exposed population and the updated exposure-response functions from epidemiological studies were fed into the IMED|HEL model. The health impacts include five kinds of health endpoints: (mortality), morbidity, number of work-loss days, health expenditures, and the value of a statistical life (Ma *et al* 2021, Zhang *et al* 2021).

## 3. Results and discussion

### 3.1. Electrification has driven high CO_2_ emission reductions

**Figure 2.**
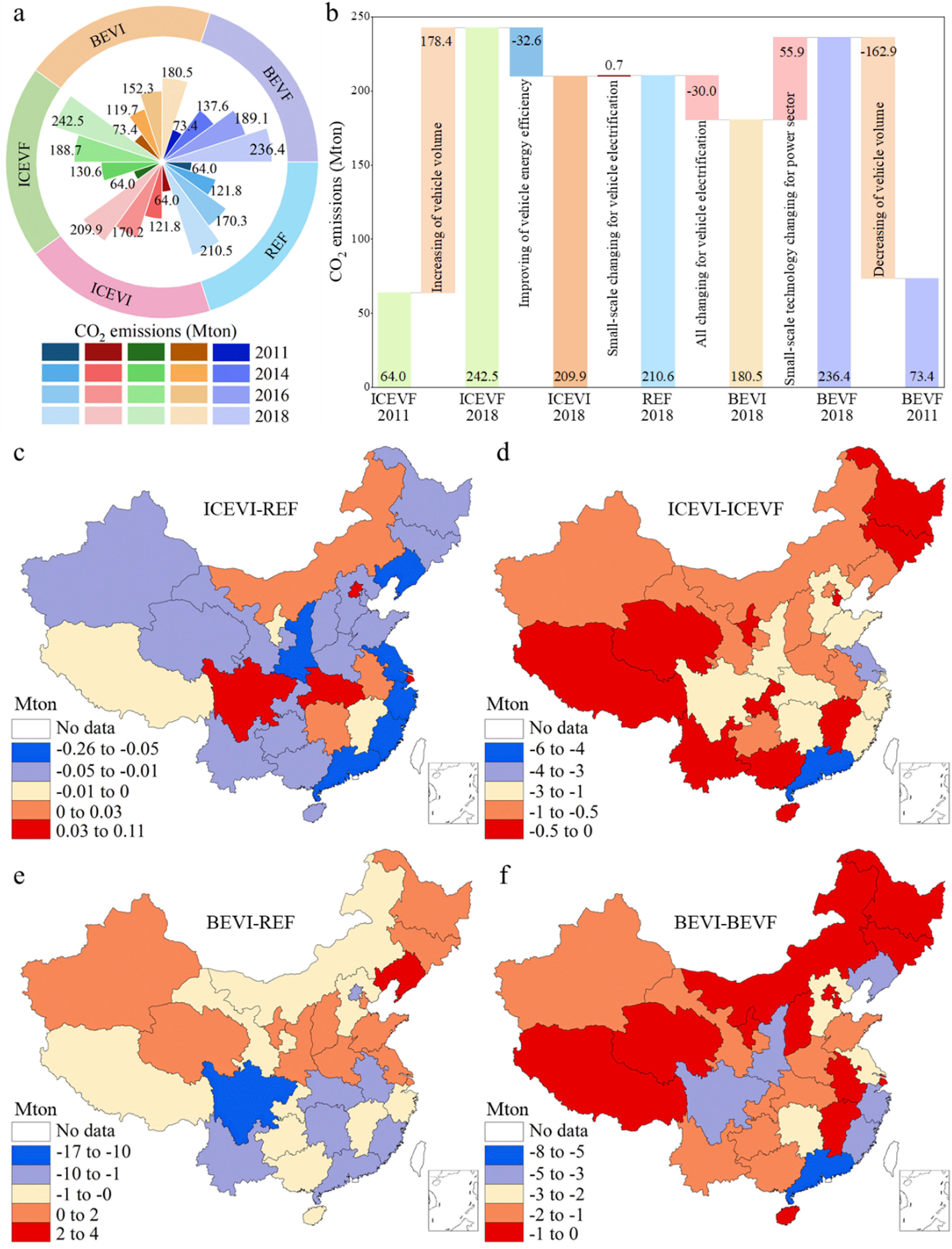
China’s private vehicle fleet caused CO_2_ emission distributions in the REF and four counterfactual scenarios. (a), Temporal distribution of CO_2_ emissions from 2011 to 2018. (b), CO_2_ emission differences among the five scenarios. (c)–(f), Spatial distribution of CO_2_ emission differences between ICEVI and REF (c), ICEVI and ICEVF (d), BEVI and REF (e), and BEVI and BEVF (f), in 2018.

Temporally, CO_2_ emissions under REF and the other four counterfactual scenarios from 2011 to 2018 all exhibited an increasing trend (figure 2(a)). The demand for transportation services generally increases with the population and economic growth (Wang *et al* 2019). Accordingly, CO_2_ emissions have risen from 64.0 Mton in 2011 to 210.5 Mton in 2018, with an average annual growth rate of 18.5% in the REF scenario. The decreasing order of CO_2_ emissions in China was ICEVF > BEVF > REF > ICEVI > BEVI in 2018.

The trend for CO_2_ emissions under the ICEVI scenario is similar to those under REF from 2011 to 2018, demonstrating that small-scale changes to vehicle structure only exert a minor effect on this type of emission. One reason is the small magnitude of the shift from EVs to ICEVs, in that the latter only declined from 82.5% in 2011 to 70.4% in 2018 (CCC 2012-2014). Therefore, vehicles fueled with high thermal electricity (BEVs, PHEVs) have similar CO_2_ emissions to ICEVs with high energy efficiency.

CO_2_ emissions under the BEVI scenario are higher than those under REF from 2011 to 2013, mainly due to the increased emissions of the power sector (Fang *et al* 2018). However, after 2013, with the power sector now slightly cleaner than in 2011, the total CO_2_ emissions for BEVI are lower than for REF, showing the high reduction potential of BEVs. CO_2_ emissions increased over time in all scenarios, mainly because of augmented vehicle stocks. Under both ICEVF and BEVF scenarios, the CO_2_ emissions respectively increased by 178.4 Mton and 162.9 Mton from 2011 to 2018 (figure 2(b)). The highest CO_2_ emissions in 2018 occurred for ICEVF, which indicates that even the power sector in BEVF is thermal-dominant, while it is CO_2_ emissions higher than in REF, it showed that EVs might not have the anticipated emission reduction effect in an unclear power system (Holland *et al* 2016); nevertheless, BEVs still offer a high emission reduction potential when compared with the frozen energy efficiency of ICEVs. Because of the energy efficiency improvement of ICEVs, CO_2_ emissions are reduced by 32.6 Mton for ICEVI compared to the ICEVF scenario in 2018. In our research, we have found that high-energy-efficient vehicles have the potential to reduce emissions significantly. Previous studies have also supported this viewpoint. The efficiency technology contributed to reducing vehicular emissions and fuel consumption (Wills and La Rovere 2010). Improving vehicle efficiency and advanced engine and transmission technology were the alternative measures for EVs, and alternative power plants were also an essential method (Atabani *et al* 2011, Mittal *et al* 2016). However, the EV promotion order in our research is mainly based on the environmental and human health impacts, without further considering the traffic congestion situation and the financial burden of EV adoption. In addition, the transport infrastructure and charging devices should also be discussed in future research. Thermal electricity decreased by 12% in BEVI relative to BEVF, leading to a 24% reduction in CO_2_ emissions in 2018 for BEVI. These results emphasize that clean electricity generation technologies are vital for PVs’ decarbonization (Williams 2012).

Spatially, we found that among all provinces, Guangdong had the highest emissions in the five scenarios, followed by Jiangsu and Zhejiang (figure S2). It is due to the high mileage per vehicle and high vehicle ownership in these provinces, both of which are almost two times the national average (MEE 2014, Feng and Xu 2016, Sun *et al* 2019). However, the increase in vehicle ownership may be associated with a decline in marginal mileage, depending on the circumstances of each individual household (Nunes *et al* 2022). For example, despite its comparable vehicle ownership to Guangdong, CO_2_ emissions are much lower in Shandong (figure S3), primarily because of the latter’s lower vehicle-traveled mileage, being almost a quarter that of Guangdong’s. Accordingly, Guangdong has the highest CO_2_ emission reductions under the ICEVI scenario relative to REF, which accounted for 40% of the total decline. By contrast, its decrease in Shandong amounted to only 6% of the total reductions in China (figure 2(c)). Emission reductions under the ICEVI scenario characterize all provincial regions because of its higher energy efficiency relative to relative to ICEVF (figure 2(d)).

BEVI scenario has the highest potential for CO_2_ emission reductions, despite having a high share of thermal power production. Spatially, the highest CO_2_ emission reduction occurred in Sichuan under BEVI (figure 2(e)), primarily due to the cleaner electricity production and its significantly more number of vehicles relative to other regions. However, the BEV adoption in Liaoning, Shaanxi, Jiangsu, Xinjiang, and Shandong led to high CO_2_ emissions under the BEVI scenario, which exceeded those under REF. The cleanness of the power sector, vehicle ownership, and traveled mileage are the significant determinants of CO_2_ emissions of BEVs (Milovanoff *et al* 2020). If BEVs develop under the context of replacing fossil-fuel-based technologies with renewables for power generation, this will substantially reduce CO_2_ emissions (Knobloch *et al* 2020).

Further, EVs, especially BEVs, are strongly compatible with high economic competitiveness and are apt to earn consumer acceptance after introducing preferential policies for buying them (Luderer *et al* 2021, Pauliuk *et al* 2021). Compared with the power production-frozen scenario (BEVF), BEVI resulted in the highest emission reductions in Guangdong (figure 2(f)), primarily because of a slightly cleaner development of electricity there (10% of thermal electricity difference between ICEVI and ICEVF in 2018). Hence, from the perspective of CO_2_ emission reductions, we find that BEVs constitute a good choice for road transport’s decarbonization as the power sector shifts towards cleaner energy production.

### 3.2. Implications for air pollutant emissions

**Figure 3.**
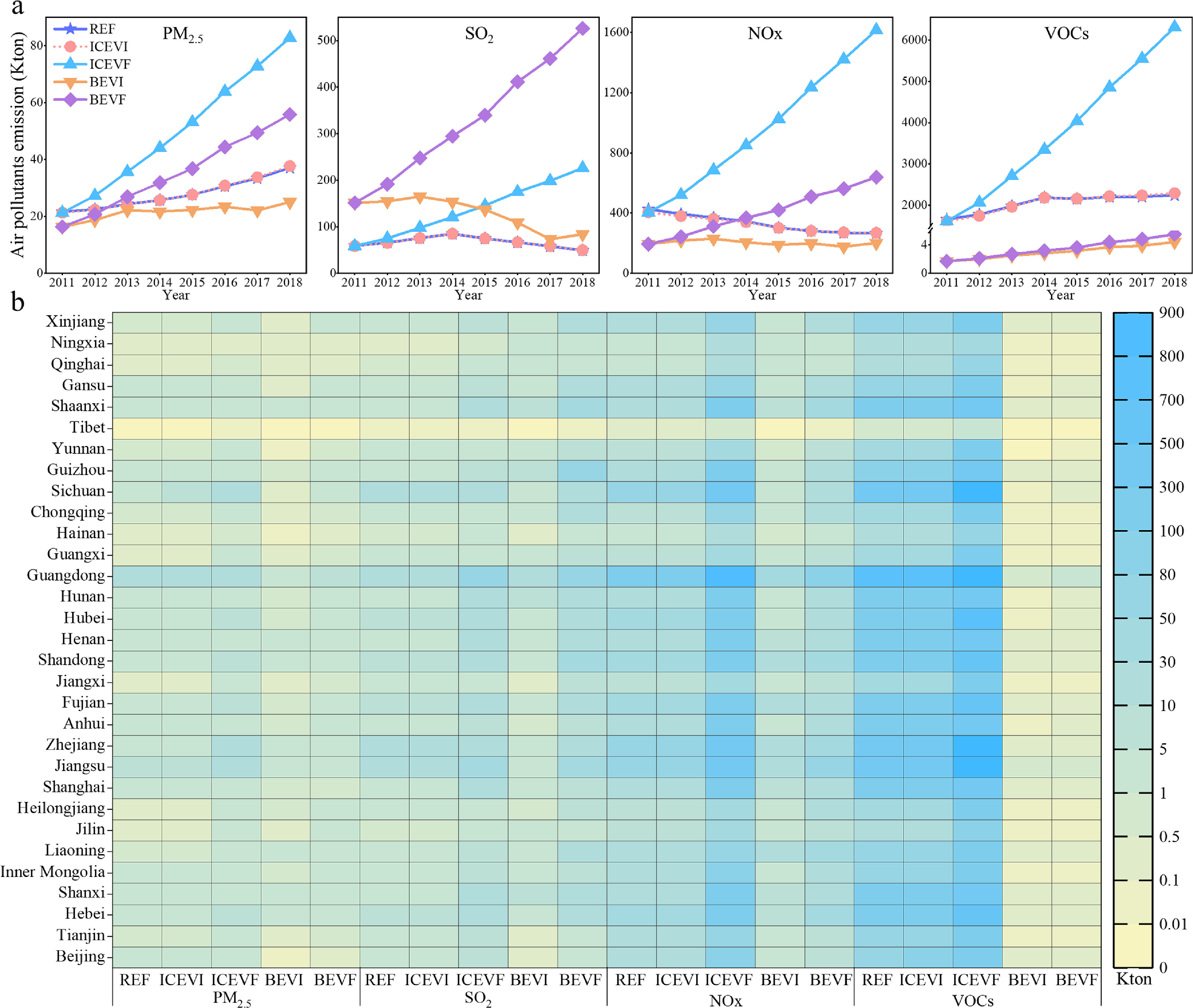
Spatiotemporal distribution of four air pollutants’ emissions under five scenarios. (a), Temporal distribution of PM_2.5_, SO_2_, NO*_X_*, and VOCs emissions from 2011 to 2018. (b), Spatial distribution of air pollutants emission in 31 provincial regions of China in 2018.

SO_2_ and VOCs emission in REF showed a trend of first increasing and then decreasing from 2011 to 2018 (figure 3(a)). The SO_2_ emission increased from 59.5 Kton in 2011 to 85.1 Kton in 2014 but declined to 49.6 Kton in 2018. However, PM_2.5_ increased continuously from 2011 to 2018 for REF, mainly because of the substantial increase in vehicle ownership over that period. Even introducing high-standard vehicles (such as the State V type) could not offset emissions from greater energy demand caused by rising vehicle ownership. The NO*_x_* emission in REF declined over time, likely influenced by the more significant usage of high-emission standard vehicles from 2011 to 2018. Both ICEVI and REF scenarios have similar emission trends for the two air pollutants from 2011 to 2018, suggesting that energy efficiency improvements and a small-scale change of technical structure (going from ICEVs to EVs) have nearly the same effects on air pollutants. The highest PM_2.5_, NO*_x_*, and VOCs emissions under five scenarios occurred for ICEVF. The potential for air pollutants emission is high from ICEVs with low energy efficiency. However, the highest SO_2_ emissions arose under the BEVF scenario, primarily due to the increased emissions from unclean power sources, which collectively supply many BEVs. The BEVI scenario had the least PM_2.5_, NO*x*, and VOCs emissions from 2011 to 2018, indicating high mitigation effects from adopting BEVs (Ke *et al* 2017). Lower air pollutant emissions are also affected by the technology improving the flue gas treatment in power generation plants (Yu *et al* 2020). The BEVI scenario has higher SO_2_ emissions relative to REF because of its higher share of thermal power (higher than 73% in 2018 (NBSC 2019b)).

Nevertheless, SO_2_ emissions diminished with time for BEVI, falling by around 45% by 2018 compared with 2011. It implies that as power plants attain cleaner operations (even at a small scale), SO_2_ emission reduction effects become significant. In addition, an SO_2_ tax could contribute to regional air pollutants reduction, which favors the renewable utilization of power sectors (Xie *et al* 2018), especially the penetration of wind power (Dai *et al* 2016, Herran *et al* 2016).

Under the REF scenario, the total electricity consumed by vehicles is small, ranging from 0.37 thousand tons of oil equivalent (Ktoe) in 2011 to 0.27 million tons of oil equivalent (Mtoe) in 2018 in China. Low electricity consumption is critical to lower SO_2_ emissions for REF. Due to the increased cost-competitiveness and a greater renewable electricity supply (Ludereret *et al* 2021), EV technologies will increase competitiveness in reducing air pollutants.

Spatially, in the five scenarios, the highest air pollutant emission levels of PM_2.5_, SO_2_, NO*x*, and VOCs occurred in Guangdong among 31 provincial regions in 2018 (figure 3(b)). Both PM_2.5_ and SO_2_ have relatively low emission levels in all provincial regions when compared those of either NO*x* or VOCs. ICEVI scenario incurred almost similar air pollutant emissions as REF across the 31 provincial areas, with the difference between these two scenarios in most regions being <0.01 Kton. By comparison, the difference in VOCs emissions between ICEVI and REF is >2 Kton in eastern China. Under the BEVI scenario, most regions have lower air pollutant emissions than under REF, and emissions are higher for ICEVF and BEVF than ICEVI and BEVI, respectively, in all of China’s mainland regions (figures S4–S7). These air pollutants emission levels show that the BEVI scenario harbors much potential for emission reductions vis-à-vis the other scenarios. It will increase with the development of cleaner power plants. Evidently, the competitiveness of BEVs is pronounced in achieving air pollutant emission reductions in China (Wang *et al* 2021).

Overall, besides the climate and geographical differences, several factors also contribute to the heterogeneity at the regional level caused by EV penetration. Firstly, the differences in the levels of socio-economic development across regions (e.g. varying population, economy, and consumption patterns) can result in variations in the technological structure and EVs promotion, thus leading to regional differences in emissions. Secondly, the carbon intensity of the provincial electricity grid affects expected carbon emission reduction, and provinces with higher carbon intensive grid could achieve higher emission reduction when gasoline vehicles are substituted with EVs. Thirdly, due to the implementation of different policies and regulations promoting EV in different regions, various incentives and barriers exist for EV development. Consequently, the penetration of EVs varies among provinces.

### 3.3. Air quality and regional health benefits

**Figure 4.**
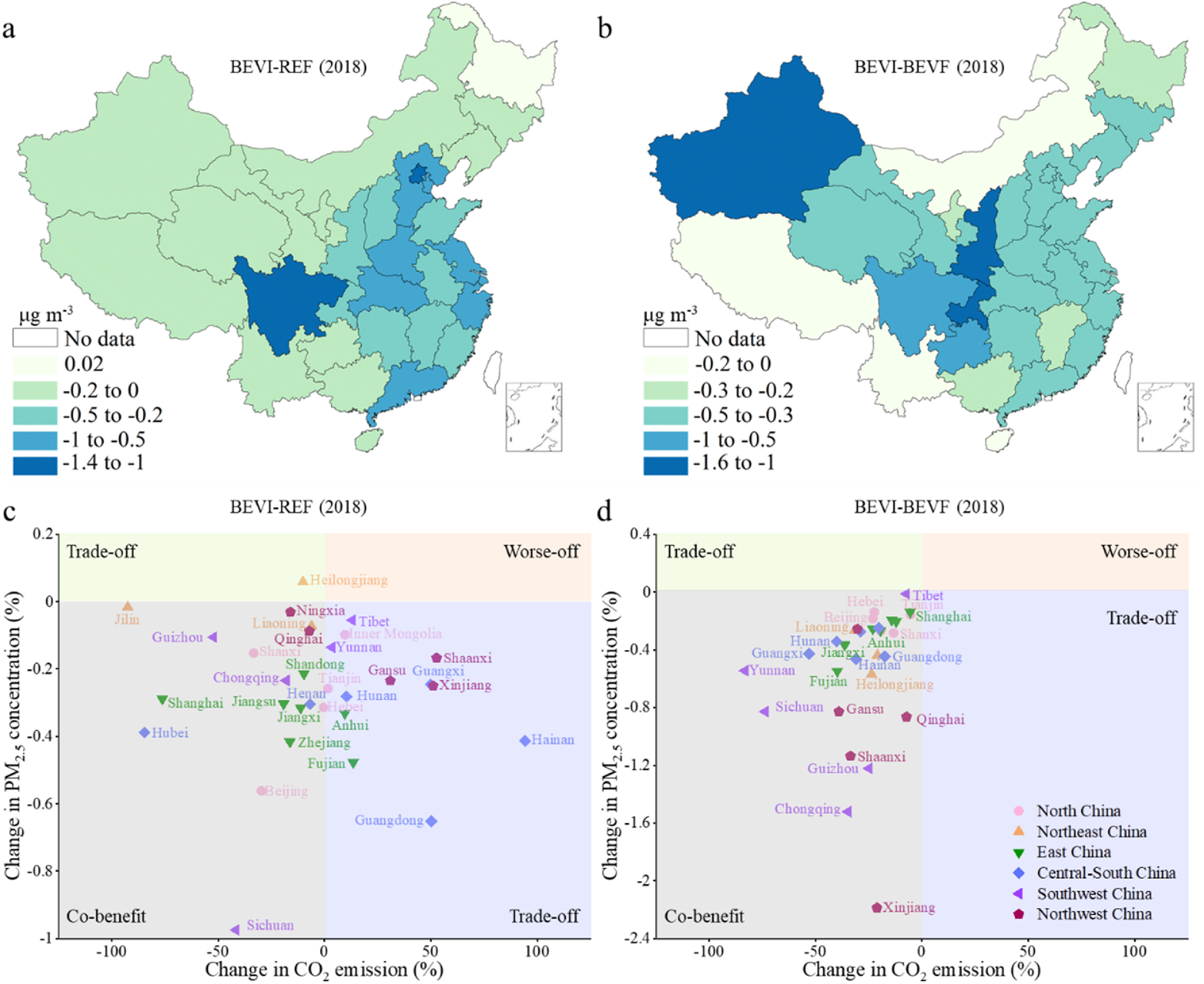
PM_2.5_ concentration changes and their co-benefits with CO_2_ emission reductions between scenarios in 2018. (a), Changes between BEVI and REF. (b), Changes between BEVI and BEVF. (c), Changes in the PM_2.5_ concentration and CO_2_ emission under the BEVI scenario relative to the REF scenario in 31 provincial regions of China. (d), Changes in the PM_2.5_ concentration and CO_2_ emission under the BEVI scenario relative to the BEVF scenario in the 31 provincial regions.

The electrification of PVs in the BEVI scenario consistently reduced the PM_2.5_ concentrations in all provincial regions of China relative to REF, except Heilongjiang, where the PM_2.5_ concentration was 0.02 *μ*g m^−3^ higher in the BEVI than REF scenario (figure 4(a)).

The extraordinary results in Heilongjiang may be due to the high primary PM_2.5_ emissions and its relatively high reliance on thermal power generation. The PM_2.5_ concentration fell in the BEVI scenario compared with REF, and the reduction ranged from 0.001 *μ*g m^−3^ in Tibet and 1.4 *μ*g m^−3^ in Beijing.

Most central and south China provincial regions have a higher avoided morbidity and mortality risk under the BEVI scenario. According to the air quality analysis, fleet electrification can readily induce more considerable abatement of the ambient PM_2.5_ concentration. Because of the slightly cleaner power development in the BEVI scenario, the air quality has improved significantly relative to the BEVF scenario (figure 4(b)). This result highlights the essential role the power sector plays in air pollutants’ mitigation by BEVs.

Some regions in western and central China undergo higher reductions (>1 *μ*g m^−3^) in their PM_2.5_ concentration in the BEVI scenario compared with BEVF. This result reveals that power plants with a slightly low share of thermal electricity will significantly influence air quality when using BEVs on the road. We also found no significant difference in PM_2.5_ concentration for the small-scale transfer of EVs to ICEVs that are highly energy efficient (i.e. the difference between REF and ICEVI scenarios) (figure S8). More than 15 provincial regions in China have substantially improved air quality under the BEVI scenario relative to REF (figure 4(c)). Taken together, and these results reveal that fleet electrification with slightly cleaner-sourced electricity will enhance air quality and decrease CO_2_ emission synergistically (figure 4(d)).

**Figure 5.**
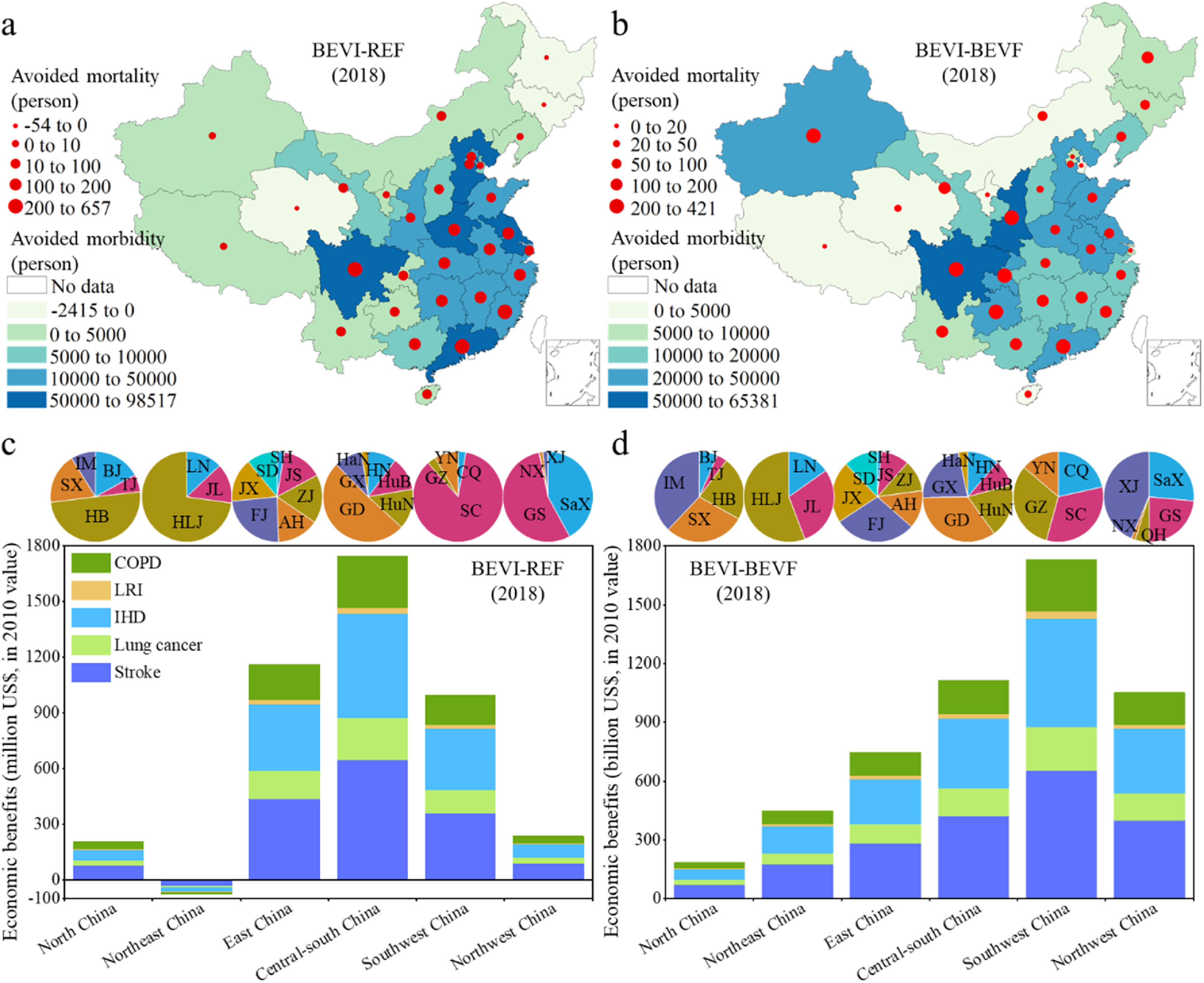
Avoided PM_2.5_-related morbidity, mortality, and health economic benefits. (a), Avoided morbidity and mortality in the BEVI scenario relative to the REF scenario in 2018. (b), Avoided morbidity and mortality in the BEVI scenario relative to the BEVF scenario in 2018. (c), Health benefits in the BEVI scenario relative to the REF scenario in 31 provincial regions of China in 2018 for stroke, lung cancer, ischemic heart disease (IHD), lower respiratory infections (LRI), and chronic obstructive pulmonary disease (COPD); along the top, the pie charts show the regional share of these health benefits for each administrative region in China. (d), Health benefits in the BEVI scenario relative to the BEVF scenario in the 31 provincial regions in 2018 with respect to the same five diseases and the corresponding regional share. The spatial distribution of China’s mainland China appears in figure S1. North China includes Beijing (BJ), Tianjin (TJ), Hebei (HB), Shanxi (SX), and Inner Mongolia (IM); Northeast China includes Liaoning (LN), Jilin (JL), and Heilongjiang (HLJ); East China includes Shanghai (SH), Jiangsu (JS), Zhejiang (ZJ), Anhui (AH), Fujian (FJ), Jiangxi (JX), and Shandong (SD); Central-South China includes Henan (HN), Hubei (HuB), Hunan (HuN), Guangdong (GD), Guangxi (GX), and Hainan (HaN); Southwest China includes Chongqing (CQ), Sichuan (SC), Guizhou (GZ), Yunnan (YN), and Tibet (TB); Northwest China including Shaanxi (SaX), Gansu (GS), Qinghai (QH), Ningxia (NX), and Xinjiang (XJ).

Under the BEVI scenario, China avoided 638 599 cases of morbidity and 3167 premature deaths in 2018 compared to the REF scenario (figure 5(a)). The most significant impact was seen in eastern China, particularly in Guangdong, where 72 216 cases of morbidity and 657 premature deaths were avoided, and Sichuan, where 98 517 cases of morbidity and 632 premature deaths were avoided. The BEVI scenario had 554 144 avoided morbidity and 3916 avoided mortalities due to a slightly cleaner power sector than under BEVF, in which the power production structure kept constant at the 2011 level until 2018. Sichuan province featured the highest avoided morbidity and mortality in the BEVI scenario relative to BEVF, having 65 381 and 421 cases, respectively (figure 5(b)).

Most central and southern China provincial regions experienced higher avoided morbidity and mortality under the BEVI than the REF scenario. We further analyzed the monetary benefits of better human health for BEVI compared with REF as well as BEVF. Large parts of China accrued health benefits because of the complete fleet electrification and slightly cleaner power (figure 5(c)). Compared with REF, the total health benefits under the BEVI scenario are US$4269 million. Under BEVI, we find considerable health benefits in central-south China of US$1744 million, of which Guangdong accounts for 51%. Yet, relative to REF, the BEVI scenario in northeast China incurred a loss of health benefits corresponding to US$75 million more in health costs. The worse-off health benefits in northeastern China are mainly due to the relatively low share of EVs and its high penetration of thermal power under the current status (i.e. REF). Thus, the greater penetration of EVs will lead to more CO_2_ and air pollutants emitted in the power sector, which induces a negative health impact in the region.

Nevertheless, slightly cleaner power will lead to overall higher health benefits in China (figure 5(d)). Southwest China has the highest health benefits of US$1732 million in BEVI relative to BEVF. The result demonstrates that clean production in the power sector is the key to realizing low emissions and health benefits during fleet electrification. ICEVs with a high energy efficiency under the ICEVI scenario will also avoid morbidity and mortality relative to ICEVF, though the health burden will rise for ICEVI versus REF (figure S9). It suggests that for health impacts, the high efficiency of ICEVs is inferior to electrification.

Our study demonstrates that the power sector significantly affects CO_2_ emission reductions and air quality improvement for BEVs utilization in China. Using BEVs and lower thermal electricity in the BEVI scenario will prevent 3167 premature deaths from PM_2.5_ exposure relative to REF in 2018. Thus, BEVs could be the best choice for the decarbonization development and air quality improvement for PVs under a cleaner power supply. However, hydrogen FCVs can also reduce CO_2_ emissions and improve air quality (Jacobson *et al* 2005, Jacobson 2008). Because our study is based on historical data for scenario design, FCVs were not considered. In addition, a previous study reported that FCVs are unnecessary for passenger vehicles and useful in freight road transportation, aviation, and navigation (Plötz 2022).

Our study also shows that enhancing the energy efficiency of ICEVs can also decrease CO_2_ and air pollutants emissions in most regions of China, indicating the potential for energy efficiency improvements for climate change mitigation (Kobayashi *et al* 2009). Meanwhile, our study uncovered a 90% higher energy efficiency of BEVs than ICEVs. Therefore, BEVs are also viable high-energy efficiency technology options for saving energy and advancing decarbonization (Chlopek *et al* 2018), which should be massively promoted in China’s passenger road transport sector.

### 3.4. Priority of BEVs considering the regional heterogeneity

**Figure 6.**
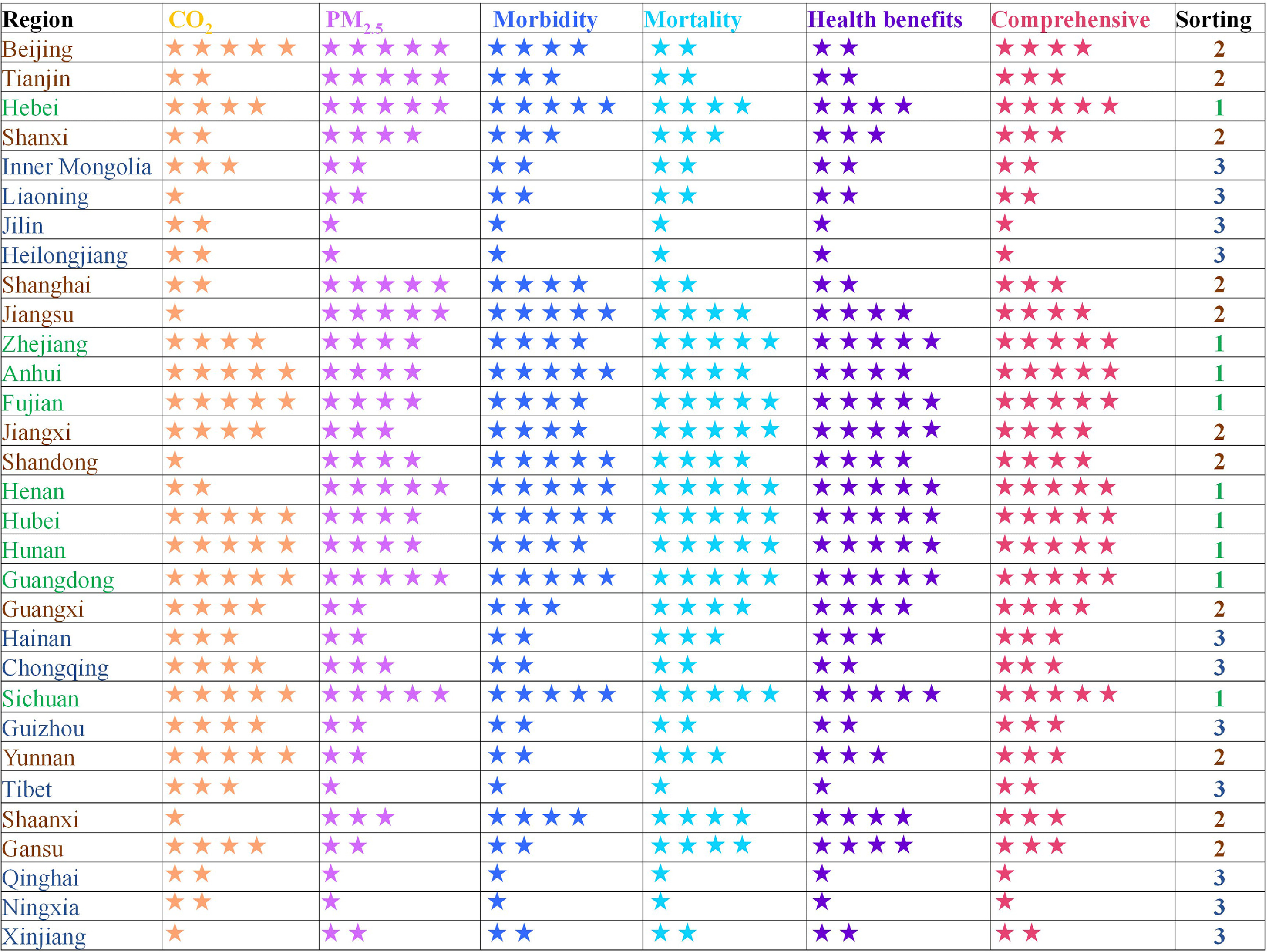
Sort ordering for electric vehicle promotion in 31 provincial regions of China according to different purposes in a holistic perspective. *Note:* Different colors for stars correspond to the promoting level of the electric vehicle; more stars indicate a higher promoting index. The numbers 1, 2, 3 in the far-right column represent the gradient sorting of the electric vehicle’s promoting level: ‘1’ is the highest level, followed by ‘2’, and then ‘3’.

This study analyzed the temporal and spatial distribution of CO_2_, air pollutants, PM_2.5_ concentrations, avoided premature deaths, and value of statistical life arising from BEVs’ penetration using retrospective analysis. Based on the holistic consideration of these parameters, we categorize three gradient scores (1, 2, 3) for promoting BEVs in the 31 provincial regions, nine of which are ranked first (i.e. =1) for promoting BEVs (figure 6). Given the thermal-dominant power sector in the current situation, we also identified eight provincial regions for developing ICEVs with a high energy efficiency under presently high thermal electricity production (table S3). Inner Mongolia, the northeast, Tibet and some areas in the northwest are appropriate regions for the high energy-efficient ICEVs promotion in the current situation. Our results showed that vehicle electrification under thermal power declined from 82.5% to 70.4% and can prevent 0.55 million cases of morbidity and ca. 3900 cases of mortality, resulting in avoided health expenditures of US$5278 billion. For the ICEV, its energy use efficiency improving by 1 Ktoe 100 km^−1^ will avoid ca. 1.39 million morbidity cases and ca. 8100 mortality cases, with related health expenditures reduced by US$10 950 billion, this accounting for almost 5.8% of China’s GDP (gross domestic product) in 2018 (NBSC 2019a). Thus, BEVs and developing highly energy-efficient ICEVs could be proposed in different provincial regions for achieving deep decarbonization and health benefits.

EVs are recognized as the most promising technology for decarbonization and health benefits for on-road transportation (Liang *et al* 2019). Yet the technology mix of power production directly impacts CO_2_ and air pollutant emissions, air quality, and the health benefits associated with EVs’ development. Our research reveals that since the current power sector still has a high share of thermal electricity, further improving the energy efficiency of ICEVs is essential for realizing climate and health benefits. This also applies to the transitional stage towards a higher percentage of EVs on the road. The burden on the climate and environment from EVs is only at the battery charging stage, which depends on the power production technology (Marczak and Drozdziel 2021). EVs’ promotion should be accelerated together with progress in the renewable energy development of the power sector (Vliet *et al* 2011). The quantitative findings of EV penetration for health impacts and its benefits presented in this study can fill the research gap and provide evidence for policy-making to develop EVs in China.

Road transport technologies electrification has the benefits of carbon and air pollutants emission reduction and human health improvement. However, EV promotion policies also exhibit several trade-offs. From the material side, the surge in demand for power batteries may increase the prices of critical battery materials, such as cobalt (Baars *et al* 2021), nickel (Elshkaki and Shen 2019), manganese (Feng *et al* 2022), and lithium (Greim *et al* 2020), for widespread EVs shortly (Wang *et al* 2023). The Ministry of Finance has allocated nearly 19 billion yuan in subsidies for constructing new energy vehicles and charging infrastructure in 2023, accounting for around 0.02% of GDP (MFPRC 2022). Financial incentives are also offered to local governments that achieve goals for installing new household chargers. Meanwhile, EV manufacturing entails a much shorter supply chain than ICEV, implying that the job-creating benefits are significantly lower than the conventional vehicles (Papoutsoglou *et al* 2022).

Several uncertainties in this research remain that should be addressed in future studies. Firstly, the air quality estimated by the GAINS model only considered the energy consumption changes in the road transport sector. Further, the simulation year used by GAINS is 2015 for estimating the results for 2011–2018 in this study. These could cause more uncertainty in the air quality evaluation. Secondly, this study focused only on PVs to reveal the full impact of vehicle electrification on road transportation transition in China, and other vehicle types should be taken into account in future studies. Moreover, the gradient sorting (rank ordering) for EV development is based on the current fleet structure and power mix, with no detailed uncertainty analysis to supplement it. Additionally, the prioritization of promoting EVs was proposed to achieve comprehensive electrification. Still, there was a lack of specific inter-provincial differentiated strategies for gradually promoting EVs. Furthermore, the order conducted from environmental and human health perspectives in our study, for future research should comprehensively consider factors such as EV production, consumption, scrappage, and infrastructure construction.

## 4. Conclusions

To rank the promotion order of EVs in 31 provincial regions of China, we chose emission reduction, air quality, human health and health benefits for assessment. The temporal and spatial heterogeneity of CO_2_ and air pollutants emissions, air quality, human health and monetized values of health benefits were quantified in our study. The results indicate that fleet electrification with cleaner power and developed regions like Guangdong can significantly reduce CO_2_ emissions and improve overall air quality. However, in high thermal power and underdeveloped provinces, such as Inner Mongolia, advancing the development of high energy-efficient ICEVs is better than adopting EVs for climate change mitigation, air quality improvement, and health benefits. Our proposed gradient levels for developing EV and ICEV can lead to significant co-benefits of reducing CO_2_ emissions and improving air quality. We can avoid morbidity and achieve numerous health benefits using these derived rankings. Our study provided a valuable reference for developing transport transitions and maximizing policies that aimed to enhance climate and health benefits, taking into account regional heterogeneity in China.

## Data Availability

The data cannot be made publicly available upon publication because they are owned by a third party and the terms of use prevent public distribution. The data that support the findings of this study are available upon reasonable request from the authors.
